# Comparative Efficacy of Minoxidil and 5‐Alpha Reductase Inhibitors Monotherapy for Male Pattern Hair Loss: Network Meta‐Analysis Study of Current Empirical Evidence

**DOI:** 10.1111/jocd.70320

**Published:** 2025-06-30

**Authors:** Aditya K. Gupta, Mary A. Bamimore, Greg Williams, Mesbah Talukder

**Affiliations:** ^1^ Mediprobe Research Inc. London Ontario Canada; ^2^ Division of Dermatology, Department of Medicine, Temerty Faculty of Medicine University of Toronto Toronto Ontario Canada; ^3^ Farjo Hair Institute London UK; ^4^ School of Pharmacy BRAC University Dhaka Bangladesh

**Keywords:** alopecia, androgenetic alopecia, male pattern baldness

## Abstract

**Background:**

Treatment options for male androgenetic alopecia (AGA) range from pharmacologic agents—such as minoxidil, finasteride, and dutasteride—to newer procedural and experimental therapies.

**Aims:**

We determined the relative effect of the various dosages and administrative routes of minoxidil, finasteride and dutasteride through network meta‐analysis (NMA) of relevant outcome measures.

**Methods:**

We conducted a systematic review to identify eligible studies. Our NMAs included studies that investigated monotherapy with minoxidil, finasteride, and dutasteride of any dosage and route on the following 5 outcomes: 24‐ and 48‐week changes in total and terminal hair density, and 24‐week change in independent observer assessment (IOA). We assessed evidence quality and performed sensitivity and node‐splitting analyses of inconsistency. Each NMA produced estimates for pairwise relative effects and surface under the cumulative ranking curve (SUCRA) values.

**Results:**

Our search found 33 eligible studies across which 19 comparators (18 interventions and 1 control) were identified. The active comparators included minoxidil (oral, topical, sublingual), finasteride (oral, topical, mesotherapy) and dutasteride (oral, mesotherapy). The control node amalgamated placebo and vehicle arms.

**Conclusions:**

We found dutasteride 0.5 mg/day to be the most effective option. Among FDA‐approved treatments, topical minoxidil 5% was the most effective topical monotherapy, while finasteride 1 mg/day was the most effective oral option. Dutasteride mesotherapy appears significantly less effective than oral administration (0.5 mg/day).

## 1 Introduction

Androgenetic alopecia (AGA) is a very common hair loss disorder in men [1, 2]. A wide range of treatments is available for male AGA, from pharmacologic agents (e.g., minoxidil, finasteride and dutasteride) to newer procedural and experimental therapies [1, 2, 3]. Finasteride and dutasteride, both 5‐α reductase inhibitors (5‐ARIs), treat male AGA by reducing dihydrotestosterone (DHT) levels, whereas minoxidil, a vasodilator, has a different mechanism in preventing hair loss [4, 5]. Among these treatments, only oral finasteride 1 mg/day and topical minoxidil (5%, 2%) have received FDA approval [2]. Recently, topical finasteride (0.25% w/w) has been approved in Italy, Germany, Portugal, Switzerland, Spain, South Korea, and Saudi Arabia. However, it has not yet been approved by the FDA in the USA or the MHRA in the United Kingdom (UK) [6]. Dutasteride (oral 0.5 mg/day) is FDA approved to treat benign prostatic hyperplasia. However, it is not approved to treat male AGA. In contrast, it is approved in Japan, Taiwan, and Korea to treat male AGA. Despite the widespread use of these therapies, direct comparative data on their efficacy remain limited.

This network meta‐analysis (NMA) aims to determine the comparative effectiveness of the various dosages and administrative routes of minoxidil, finasteride, and dutasteride in AGA.

## 2 Methods

The protocol for the current network meta‐analysis (NMA) study was prospectively registered in the *International Platform of Registered Systematic Review and Meta‐analysis Protocols* under the ID “INPLASY202540076”. The *Preferred Reporting Items for Systematic reviews and Meta‐Analyses* (PRISMA) extension for NMAs guided the entire conduct of our work [7].

As alluded to, the goal of our work was to use currently existing empirical data to quantitatively determine the relative effect of monotherapy with minoxidil, finasteride and dutasteride in male pattern hair loss. As per the PICO (i.e., *Patient*, *Intervention(s)*, *Comparator(s)*, and *Outcome(s)*) framework: *Patient* corresponded to males diagnosed with androgenetic alopecia (AGA) [8]. *Intervention* corresponded to monotherapy with any dosage of minoxidil, finasteride or dutasteride administered through any route (i.e., intervention of interest) while *Comparators* could be non‐active (i.e., placebo or vehicle) or active (i.e., intervention(s) of interest).

Studies that were eligible for our NMAs satisfied our PICO framework in addition to being: (1) prospective (i.e., randomized or non‐randomized) and (2) published in English. *Outcomes* were multiple, but the two primary outcomes of interest were 24‐week change in total and terminal hair density. Secondary outcomes of interest were 24‐week change in independent observer assessment (IOA) and 48‐week change in total and terminal hair density. Throughout our analyses, hair density was measured in hairs per square centimeter (hairs/cm^2^) and doses of oral interventions were quantified in milligrams (mg).

Eligible studies were identified by systematically searching the peer‐reviewed literature in PubMed and Scopus. The “Deduplicator” feature of the “Systematic Review Accelerator” (SRA) software was used to remove duplicate records; *Rayyan* software was used to manage screening of titles and abstracts, as well as full texts [9, 10]. The searches and screenings were performed independently by two authors (MT and MAB). Discrepancy was resolved through consultation with a third author (AKG). Extracted data were managed using spreadsheets and *RStudio* software was used for all quantitative analyses; the *gemtc* and *multinma* R packages were used [11].

An NMA was performed for each outcome where each outcome corresponded to a network. A network is visually depicted as a graph of nodes and edges, where a node is represented by a vertex and an edge is represented by a line between 2 nodes (i.e., two vertices). The node represents the intervention and the unit of analysis thereof. For the current NMA, nodes were defined at the level of dose such that dose and “regimen” are used interchangeably herein. Each edge in a network graph represents two nodes whose effect(s) were compared directly (i.e., compared in an actual head‐to‐head trial). For each outcome, a random‐effects Bayesian NMA was conducted with uniform priors, 4 Markov Chain Monte Carlo (MCMC) chains, 200 000 iterations, and 5000 adaptations. We conducted node splitting analyses of inconsistency for primary outcome measures.

For each outcome, we estimated regimens' pairwise relative effects and surface under the cumulative ranking curve (SUCRA) values. All outcomes, except 24‐week change in IOA, were metricized using the mean difference (MD). We used the odds ratio (OR) to metricize 24‐week change in IOA. A 95% credible interval (CI) was estimated for each MD and OR. An intervention's SUCRA value, which ranges from 0 to 1, or 0% to 100% (inclusive), is a ranking metric for its relative effect. For all quantitative analyses, alpha (α), which is the threshold for statistical significance, was set to 5% (or 0.05). Evidence quality, for each included study, was assessed using tools freely available under the Cochrane Collaboration. For sensitivity analyses, we conducted two network meta‐regressions where one (ecologically) adjusted for variation in age and the other adjusted for variation in disease severity.

## 3 Results

**FIGURE 1:**
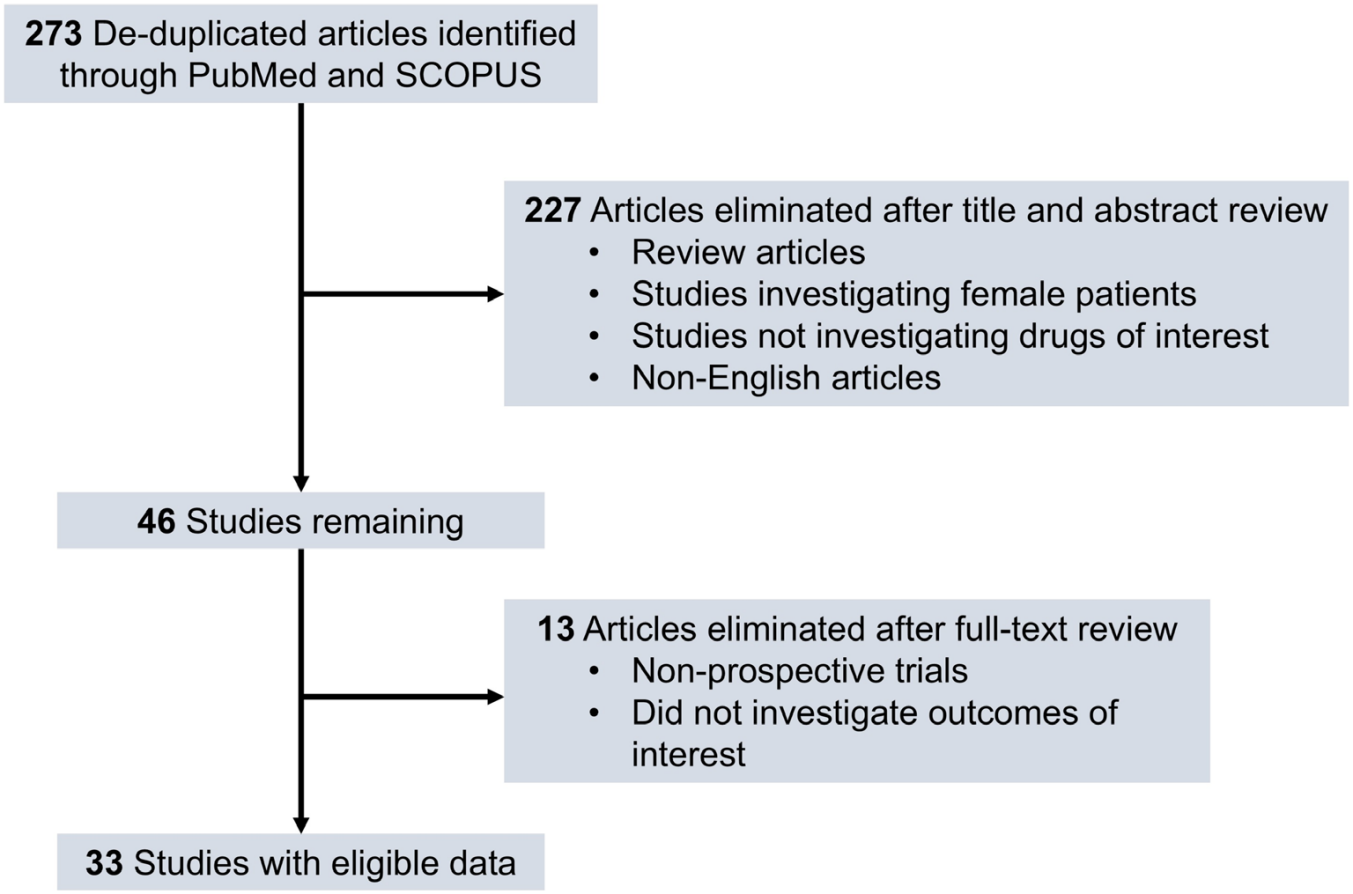
Identification of included studies. This diagram shows the systematic search process used to find studies included in the network meta‐analyses.

**FIGURE 2:**
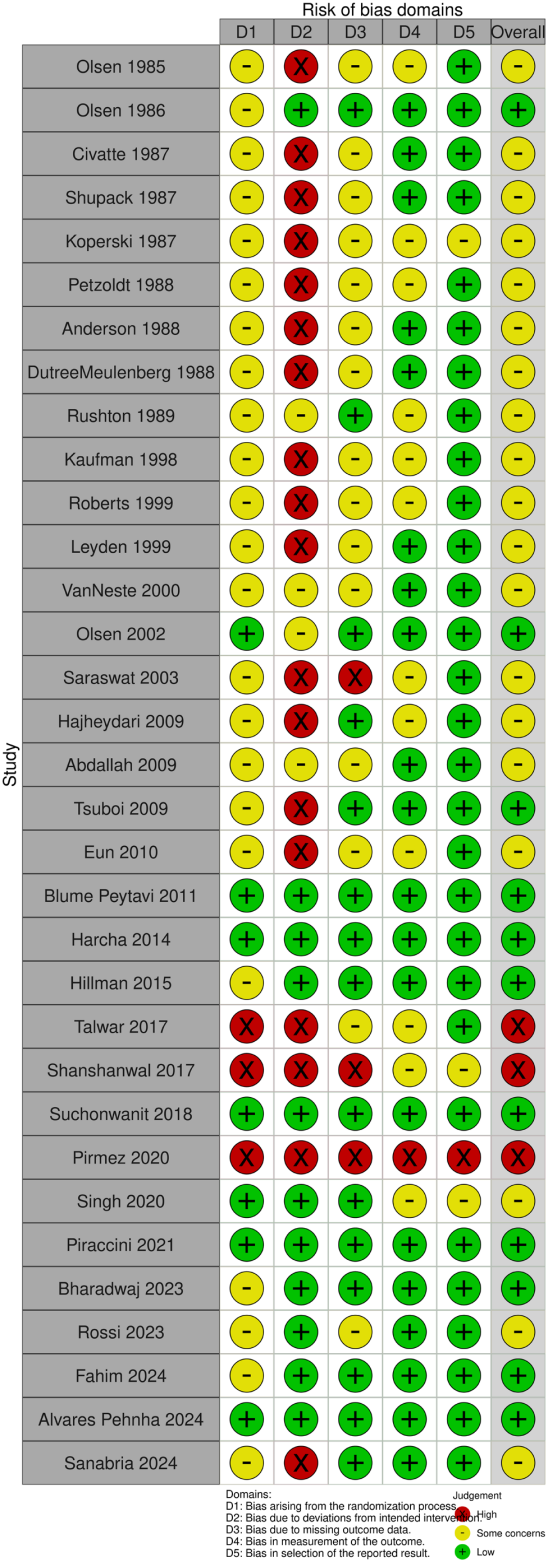
Assessment of evidence quality. This graph is a qualitative summary of the included studies' risk of bias (RoB) as per the Cochrane Collaboration's RoB tools.

**FIGURE 3:**
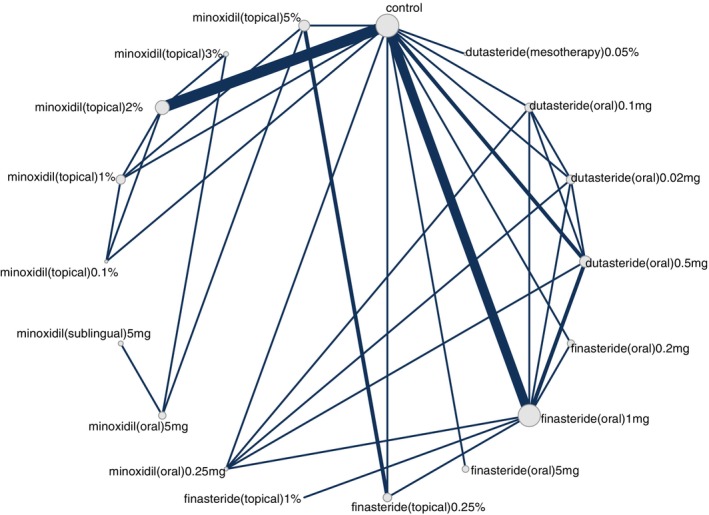
Network plot for 24‐week change in total hair density. This graph depicts regimens whose efficacy was directly compared in head‐to‐head trials for androgenetic alopecia insofar as change in total hair density at 24 weeks.

**FIGURE 4:**
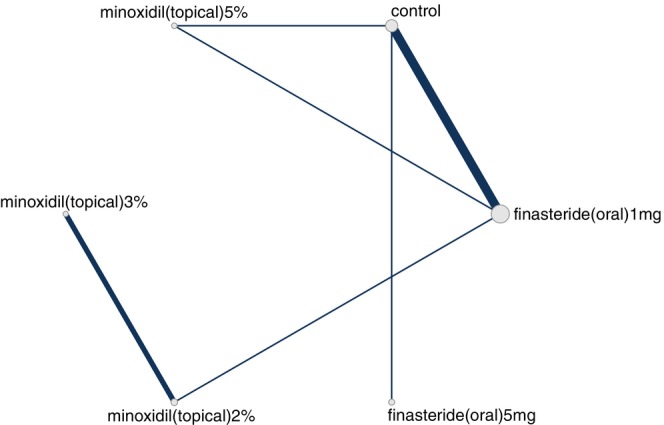
Network plot for 24‐week change in total hair density. This graph depicts regimens whose efficacy was directly compared in head‐to‐head trials for androgenetic alopecia insofar as change in total hair density at 24 weeks.

**FIGURE 5:**
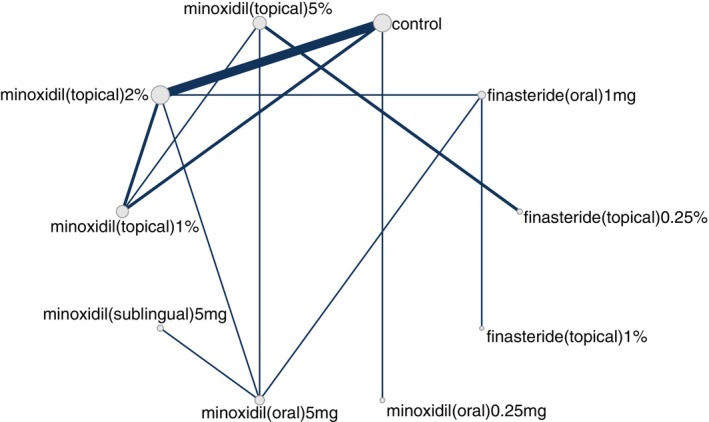
Network plot for 48‐week change in total hair density. This graph depicts regimens whose efficacy was directly compared in head‐to‐head trials for androgenetic alopecia insofar as change in total hair density at 48 weeks.

**FIGURE 6:**
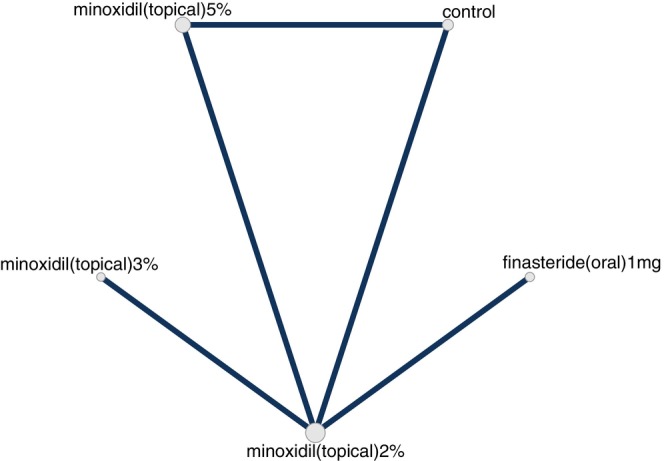
Network plot for 24‐week change in terminal hair density. This graph depicts regimens whose efficacy was directly compared in head‐to‐head trials for androgenetic alopecia insofar as change in terminal hair density at 24 weeks.

**FIGURE 7:**
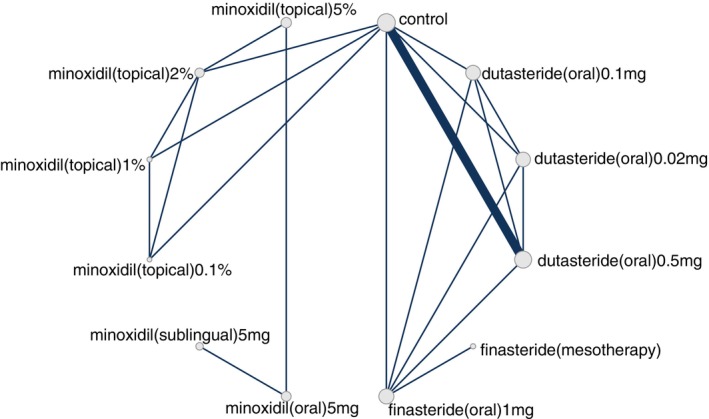
Network plot for 48‐week change in terminal hair density. This graph depicts regimens whose efficacy was directly compared in head‐to‐head trials for androgenetic alopecia insofar as change in terminal hair density at 48 weeks.

**FIGURE 8:**
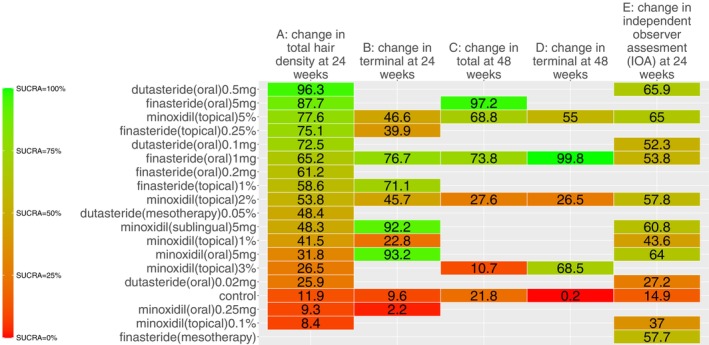
Efficacy ranking. This Kilim plot depicts regimens' surface under the cumulative ranking curve (SUCRA) values across all outcomes of interest. The congruence of the color gradient across each column (i.e., each outcome) is a barometer for how similar a regimen performs across the respective outcome measures. Values towards the green end of the spectrum represent higher SUCRA values and values towards the red end of the spectrum represent lower SUCRA values. The red and green colors were arbitrarily chosen.

**FIGURE 9:**
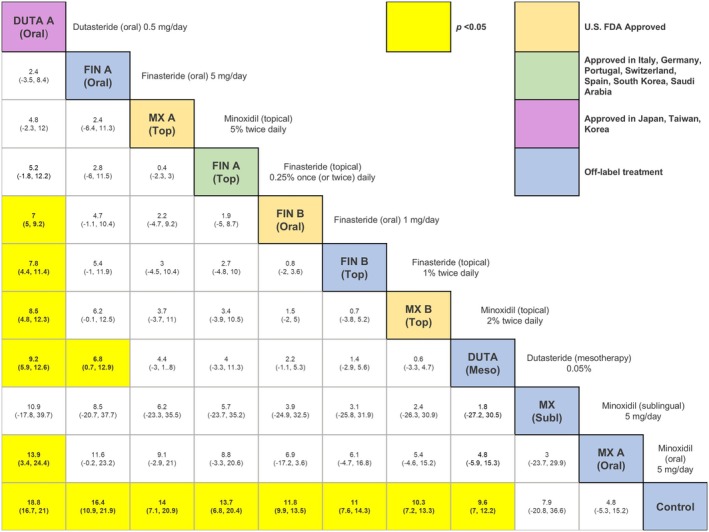
League table for 24‐week change in total hair density (10 interventions plus one control). This figure shows the pairwise comparisons of selected treatments in the largest network. The results are given as mean differences (MD) with 95% credible intervals (CI) in brackets. The control group refers to placebo or vehicle.

Our search identified 33 studies whose data were used across the 5 outcomes of interest (Figure 1). An exception was made by including data from the study by Blume‐Peytavi et al. (2011) [12]. This trial, which studied female participants with pattern hair loss, was included to allow for a connected network for the 24‐week change in IOA outcome. Study‐level summary of the included trials' characteristics is presented in Table 1 [12, 13, 14, 15, 16, 17, 18, 19, 20, 21, 22, 23, 24, 25, 26, 27, 28, 29, 30, 31, 32, 33, 34, 35, 36, 37, 38, 39, 40, 41, 42, 43, 44]. Each study's risk of bias is presented in Figure 2. The network plot corresponding to the five outcomes are in Figures 3, 4, 5, 6, 7. The kilim plot [45] in Figure 8 presents regimens' (i.e., interventions') SUCRA values for all 5 outcome measures. Regimens' pairwise relative effects, from the largest network (i.e., change in total hair count at 24 weeks), are presented in the league table in Figure 9.

**TABLE 1:** Summary of included studies' characteristics.

Study	First author (last name) and publication year	Mean age (in years); standard deviation	Active comparator(s)^a^
1	Olsen 1985	36.2; 6.05	Minoxidil (topical) 3% twice daily Minoxidil (topical) 2% twice daily
2	Olsen 1986	36.0; 5.89	Minoxidil (topical) 2% twice daily Minoxidil (topical) 1% twice daily Minoxidil (topical) 0.1% twice daily
3	Civatte 1987	34.09; 6.56	Minoxidil (topical) 2% twice daily
4	Shupack 1987	32.91; 5.62	Minoxidil (topical) 2% twice daily Minoxidil (topical) 1% twice daily Minoxidil (topical) 0.1% twice daily
5	Koperski 1987	38.3; 7.5	Minoxidil (topical) 3% twice daily Minoxidil (topical) 2% twice daily
6	Petzoldt 1988	32.9; 8	Minoxidil (topical) 2% twice daily
7	Anderson 1988	32.8; 7.5	Minoxidil (topical) 2% twice daily
8	Dutree‐Meulenberg 1988	34.3; 7.5	Minoxidil (topical) 2% twice daily
9	Rushton 1989	Range (in years): 18 to 49	Minoxidil (topical) 2% twice daily
10	Kaufman 1998	32.50; 1.31	Finasteride (oral) 1 mg once daily
11	Roberts 1999	30; 0.38	Finasteride (oral) 5 mg once daily Finasteride (oral) 1 mg once daily Finasteride (oral) 0.2 mg once daily
12	Leyden 1999	32.51; 0.64	Finasteride (oral) 1 mg once daily
13	VanNeste 2000	29.75; 0.75	Finasteride (oral) 1 mg once daily
14	Olsen 2002	36.44;6.43	Minoxidil (topical) 5% twice daily Minoxidil (topical) 2% twice daily
15	Saraswat 2003	28.38; 0.85	Minoxidil (topical) 2% twice daily Finasteride (oral) 1 mg once daily
16	Hajheydari 2009	22.8; 3.3	Finasteride (topical) 1% gel twice daily Finasteride (oral) 1 mg once daily
17	Abdallah 2009	30.82; 8.21	Dutasteride (mesotherapy) 0.05%
18	Tsuboi 2009	40.6; 6.64	Minoxidil (topical) 5% twice daily Minoxidil (topical) 1% twice daily
19	Eun 2010	38.10; 6.83	Dutasteride (oral) 0.5 mg once daily
20	Blume‐Peytavi 2011	49.9 (range (in years): 23 to 75)	Minoxidil (topical) 5% twice daily Minoxidil (topical) 2% twice daily
21	Harcha 2014	38.50; 7.80	Dutasteride (oral) 0.5 mg once daily Dutasteride (oral) 0.02 mg once daily Dutasteride (oral) 0.1 mg once daily
22	Hillman 2015	43.5; 11.67	Minoxidil (topical) 5% twice daily
23	Talwar 2017	Range (in years): 31 to > 51	Finasteride (mesotherapy)
24	Shanshanwal 2017	27.85; 5.5	Dutasteride (oral) 0.5 mg once daily Finasteride (oral) 1 mg once daily
25	Suchonwanit 2018	41.78; 12.30	Minoxidil (topical) 3% twice daily
26	Pirmez 2020	36.7 (range (in years): 23 to 35)	Minoxidil (oral) 0.25 mg once daily
27	Singh 2020	26.15; 4.16	Minoxidil (topical) 5% twice daily
28	Piraccini 2021	32.19; 5.22	Finasteride (topical) 0.25% once daily
29	Bharadwaj 2023	26.23 (range (in years): 10 to 37)	Finasteride (topical) 0.25% twice daily Minoxidil (topical) 5% twice daily
30	Rossi 2023	24.37; 2.49	Finasteride (topical) 0.25% once daily Minoxidil (topical) 5% twice daily
31	Fahim 2024	32.05; 7.05	Minoxidil (topical) 5% twice daily
32	Alvares Pehnha 2024	36.55; 7.76	Minoxidil (topical) 5% twice daily Minoxidil (oral) 5 mg once daily
33	Sanabria 2024	36.3; 9.18	Minoxidil (sublingual) 5 mg once daily Minoxidil (oral) 5 mg once daily

*Note:* The summary of the included studies' characteristics presented herein is study‐level.

Abbreviation: mg, milligram.

^a^ Only the arms of active comparators that were compared in the current study are presented.

Across the 33 studies, 19 comparators (18 interventions/1 control) were identified, namely,

- Dutasteride (oral) 0.5 mg once daily.
- Dutasteride (oral) 0.1 mg once daily.
- Dutasteride (oral) 0.02 mg once daily.
- Dutasteride (mesotherapy) 0.05%.
- Finasteride (oral) 5 mg once daily.
- Finasteride (oral) 1 mg once daily.
- Finasteride (oral) 0.2 mg once daily.
- Finasteride (topical) 1% gel twice daily.
- Finasteride (topical) 0.25% once or twice daily.
- Finasteride (mesotherapy).
- Minoxidil (oral) 5 mg once daily.
- Minoxidil (oral) 2.5 mg once daily.
- Minoxidil (sublingual) 5 mg once daily.
- Minoxidil (topical) 5% twice daily
- Minoxidil (topical) 3% twice daily.
- Minoxidil (topical) 2% twice daily
- Minoxidil (topical) 1% twice daily.
- Minoxidil (topical) 0.1% twice daily.
- Control.

The control node amalgamated outcome data from placebo and vehicle arms. NMAs were done at the level of the dosage with the exception of finasteride (topical) 0.25% once daily and finasteride (topical) 0.25% twice daily; which are combined into one node, namely, “finasteride (topical) 0.25% once or twice daily”. Our reason for this exception was based on clinical opinion and critical review of the outcome data produced by Piraccini et al. (2021), Bharadwaj et al. (2023) and Rossi et al. (2023): it was deemed clinically relevant and intuitive to amalgamate the effect of finasteride (topical) 0.25% once daily and finasteride (topical) 0.25% twice daily into one node.

The rank of effectiveness of the regimens, insofar as the 4 outcome measures related to hair density, was somewhat similar across networks, as evidenced by the congruent color gradients (Figure 8). For the 24‐week change in total hair density, dutasteride (oral) 0.5 mg once daily was the most effective (SUCRA = 96.3%) and it was significantly more effective than other regimens, including dutasteride (mesotherapy) 0.05% (MD = 9.2 hairs/cm^2^, 95% CI: (5.9,12.6) hairs/cm^2^, *p <* 0.05) (Figure 9). However, this highest‐ranked intervention was not significantly different from minoxidil (sublingual) 5 mg once daily (MD = 10.9 hairs/cm^2^, 95% CI: (−17.8, 39.7) hairs/cm^2^, *p ≥* 0.05) (Figure 9). In terms of 24‐week change in terminal hair density, minoxidil (oral) 5 mg once daily was ranked the most effective (SUCRA = 93.2%), followed by minoxidil (sublingual) 5 mg once daily (SUCRA = 92.2%) (Figure 8). These top two were not significantly different from each other (MD = −2.3 hairs/cm^2^, 95% CI: (−24.1,19.5) hairs/cm^2^, *p ≥* 0.05) as per this outcome measure (Figure S1). For 24‐week change in IOA, the highest‐ranked modality was dutasteride (oral) 0.5 mg once daily, which was only significantly more effective than control (OR = 13.5, 95% CI: 1.1, 492.7, *p ≥* 0.05) (Figure S1).

Node‐splitting analyses for inconsistency was only possible for the two primary networks, namely, 24‐week change in total and terminal hair density (Tables 2 and 3). Results from node splitting analyses support that each primary network has statistical consistency. Node splitting analyses was not possible for other outcomes because of the geometry of their network graph.

**TABLE 2:** Node‐splitting analyses for inconsistency for network corresponding to change in total hair density at 24 weeks.

Comparator 1	Comparator 2	*p*
Dutasteride (oral)0.5 mg	Finasteride (oral)1 mg	0.030075
Dutasteride (oral)0.5 mg	Control	0.012525
Finasteride (oral)1 mg	Finasteride (topical)0.25%	0.823125
Finasteride (oral)1 mg	Control	0.0013
Finasteride (topical)0.25%	Minoxidil (topical)5%	0.9169
Finasteride (topical)0.25%	Control	0.999425
Minoxidil (oral)5 mg	Minoxidil (topical)3%	0.45415
Minoxidil (oral)5 mg	Minoxidil (topical)5%	0.44825
Minoxidil (topical)1%	Minoxidil (topical)2%	0.689125
Minoxidil (topical)1%	Minoxidil (topical)5%	0.894525
Minoxidil (topical)1%	Control	0.981125
Minoxidil (topical)2%	Minoxidil (topical)3%	0.445575
Minoxidil (topical)2%	Control	0.4194
Minoxidil (topical)5%	Control	0.319175

*Note:* For comparisons whose *p*‐value is 0.05 or greater, we fail to reject the null hypothesis (where the null hypothesis states that there is no inconsistency).

**TABLE 3:** Node‐splitting analyses for inconsistency for network corresponding to change in terminal hair density at 24 weeks.

Comparator 1	Comparator 2	*p*
Minoxidil (oral)5 mg	Minoxidil (topical)2%	0.829975
Minoxidil (oral)5 mg	Minoxidil (topical)5%	0.79345
Minoxidil (topical)1%	Minoxidil (topical)2%	0.837075
Minoxidil (topical)1%	Minoxidil (topical)5%	0.83295
Minoxidil (topical)1%	Control	0.8177

*Note:* For comparisons whose *p*‐value is 0.05 or greater, we fail to reject the null hypothesis (where the null hypothesis states that there is no inconsistency).

**FIGURE 10:**
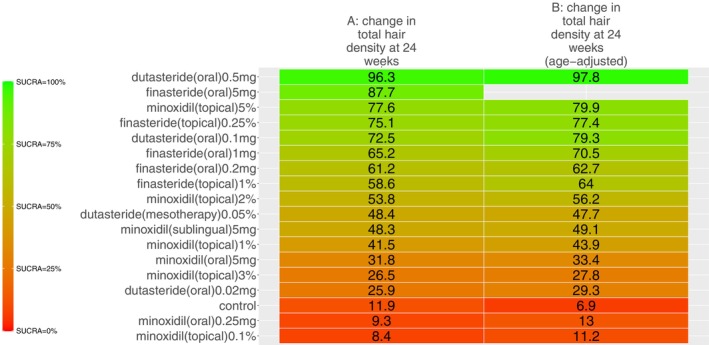
Sensitivity analyses I. This Kilim plot presents regimens' efficacy ranks for the base and age‐adjusted network meta‐analyses for 24‐week change in total hair density.

**FIGURE 11:**
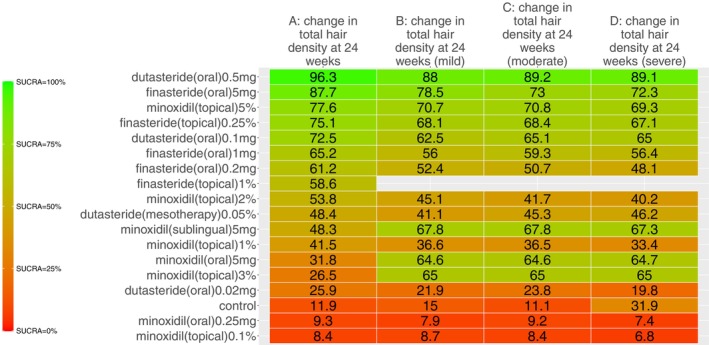
Sensitivity analyses II. This Kilim plot presents regimens' efficacy ranks for the base and severity‐adjusted network meta‐analyses for 24‐week change in total hair density.

We also used kilim plots to present results of our sensitivity analyses (Figures 10 and 11). Sensitivity analyses were only conducted for the largest network (i.e., 24‐week change in total hair density). Figure 10 shows congruent regimen SUCRA ranks for base NMA and age‐adjusted NMA. Similarly, Figure 11 shows congruent regimen SUCRA ranks for base NMA and severity‐adjusted NMA. Findings from our network meta‐regressions for age and disease severity support that findings from the base NMAs are robust.

## 4 Discussion

### 4.1 Minoxidil Versus Finasteride Versus Dutasteride

Our network meta‐analysis demonstrates that oral dutasteride 0.5 mg/day is significantly more effective than oral finasteride 1 mg/day and oral minoxidil 5 mg/day in the treatment of male AGA (Figure 9). Figure 9 displays the league table highlighting the 11 most relevant comparators (10 interventions plus one control). The league table for the 24‐week change in total hair density across 18 comparators is shown in Figure S1.

Multiple clinical studies and network meta‐analyses have corroborated the superior efficacy of dutasteride 0.5 mg/day over finasteride 1 mg/day [35, 46]. Dutasteride inhibits both type I and type II 5α‐reductase, resulting in a 92% reduction in serum dihydrotestosterone (DHT), compared to a 73% reduction with finasteride [2]. Dutasteride 0.5 mg/day and finasteride 1 mg/day share a similar safety profile, with both associated with sexual dysfunction and neuropsychiatric side effects [2, 32, 35]. Oral finasteride remains the most effective monotherapy for AGA that is FDA approved. Oral minoxidil 5 mg/day presents a higher risk of systemic adverse effects, such as hypertrichosis, compared to its topical counterpart (topical minoxidil 5% twice daily) [42].

### 4.2 Oral Minoxidil Versus Topical Minoxidil Versus Sublingual Minoxidil

This NMA suggests that minoxidil (oral 5 mg/day, topical 5% twice a day, topical 2% twice a day, and sublingual 5 mg once daily) has comparable efficacy in the treatment of male AGA. A 24‐week randomized trial found oral minoxidil (5 mg/day) and topical minoxidil (5% twice daily) produced similar improvements in terminal hair density, though oral minoxidil 5 mg/day showed superior vertex‐area results in photographic analysis [42]. Minoxidil 5% remains the most effective topical FDA‐approved monotherapy for male AGA. Notably, Olsen and colleagues showed that 5% topical minoxidil, applied twice daily, was more effective than 2% topical minoxidil (twice daily) in direct comparisons [26]. However, this trend was not reflected in our NMA, likely because it incorporated both direct and indirect comparisons. Small sample sizes in some studies may have further diminished statistical power.

Another study reported that sublingual minoxidil 5 mg/day achieved equivalent clinical improvement rates to oral minoxidil 5 mg/day for 24 weeks (42% vs. 40% vertex improvement, *p* = 0.69) [43]. Regarding safety, oral minoxidil 5 mg/day poses greater systemic risks, with hypertrichosis occurring in approximately 49% of users compared to 25% for topical formulations (topical minoxidil 5%, twice daily) (*p* = 0.02) [42]. Topical minoxidil 5%, twice daily predominantly causes localized irritation or hair shedding [42]. Sublingual administration 5 mg/day appears safer than oral minoxidil, 5 mg/day, with reduced cardiovascular effects (0% vs. 9% palpitations, *p* = 0.048) [43].

### 4.3 Oral Finasteride Versus Topical Finasteride

This NMA indicates that oral finasteride 1 mg/day and topical finasteride 0.25% demonstrate comparable efficacy in treating male AGA, a finding supported by multiple clinical studies [28, 38]. A 24‐week randomized trial found topical finasteride 0.25% w/w solution, 1–4 sprays (50–200 μL) increased target area hair count (1 cm^2^ circular area) by 20.2 hairs versus 21.1 hairs for oral finasteride 1 mg/day, indicating equivalent effectiveness. A separate study also demonstrated that the topical formulation finasteride 0.25%, twice daily reduced plasma dihydrotestosterone (DHT) by 68%–75% compared to 62%–72% for oral finasteride (1 mg/day) after 1 week of treatment, suggesting localized potency (*p* ≥ 0.11) [47]. Additionally, a 2022 study of 45 men reported both forms, finasteride oral 1 mg/day and finasteride gel 1% twice daily significantly increased terminal hair counts and reduced bald spots in a comparable way [28]. Although topical finasteride (0.25% w/w solution) resulted in less systemic DHT suppression, its clinical outcomes for hair regrowth were statistically equivalent to those of oral finasteride 1 mg/day [38]. Moreover, topical finasteride was associated with a lower likelihood of systemic side effects, such as sexual dysfunction [6, 38].

### 4.4 Oral Dutasteride Versus Dutasteride Mesotherapy

The NMA suggests that oral dutasteride 0.5 mg/day is significantly more efficacious than dutasteride mesotherapy at 0.05%. While oral dutasteride 0.5 mg/day is associated with risks of sexual side effects and psychiatric side effects [2, 48], mesotherapy 0.01% dutasteride, every 3 months predominantly resulted in localized pain (reported in 45.5% of cases) but minimal systemic absorption [49]. Mesotherapy with dutasteride is not FDA approved with no standardized North American protocol.

### 4.5 Standardization of Treatments

The NMA provides an overview of the relative effectiveness of minoxidil (oral, topical, and sublingual), finasteride (oral, topical, mesotherapy), and dutasteride (oral, mesotherapy). If there is a proliferation of generic preparations, the efficacy experienced by the end‐user may vary. Currently, there is a lack of standardization for topical finasteride in North America. However, an approved topical finasteride preparation is available in some European countries [38]. Mesotherapy protocols with finasteride and dutasteride would benefit from standardization.

### 4.6 Finasteride or Dutasteride When Planning Conception

#### 4.6.1 Finasteride

Finasteride is likely safe for use when the partner is trying conceive, as semen levels after taking 5 mg/day are well below those causing fetal harm [50]. Men taking finasteride 1 mg dose can likely continue treatment while their partners try to conceive. However, those with fertility concerns should consult a specialist and may consider discontinuing use. While finasteride has a short half‐life and is cleared from the body in a few days, DHT levels—which influence fertility—may take up to 90 days or more to normalize after stopping the medication [50]. Since there is no established timeframe for discontinuation, physicians/patients should consult current guidelines, package inserts, and local authoritative sources. Further research is needed to confirm the long‐term reproductive safety of finasteride. Finasteride 1 mg/day (but not 5 mg/day) is FDA approved for AGA.

#### 4.6.2 Dutasteride

Dutasteride in semen is highly protein‐bound, limiting vaginal absorption and posing minimal risk to a pregnant partner [50]. Even at peak levels, exposure would be far below doses linked to birth defects in animal studies. However, dutasteride may impair male fertility by reducing sperm count and motility [50]. Men trying to conceive should consider stopping dutasteride if pregnancy is delayed or before fertility evaluation. Due to its long half‐life, dutasteride can remain in the body for up to 6 months after stopping, so early discontinuation is advisable when planning conception [50]. However, physicians/patients should consult current guidelines, package inserts, and local authoritative sources prior to make any clinical decisions. Dutasteride is not FDA‐approved for treating AGA.

### 4.7 Limitations

Our NMA restricted the analysis to randomized controlled trials (RCTs) only, thereby excluding non‐controlled studies and case reports that may provide additional real‐world insights or data. Furthermore, the analysis focused solely on monotherapies, omitting combination therapies that may be commonly used in clinical practice and potentially be more effective. As a result, the findings may not fully reflect current therapeutic strategies. Additionally, as more RCTs are reported over time, the database will expand to include a larger pool of participants, which may alter or refine the conclusions drawn in this analysis.

### 4.8 Prescription and Monitoring of Treatments

For the safety of the end‐user, minoxidil (oral, sublingual), finasteride (oral, topical, mesotherapy) and dutasteride (oral, mesotherapy) must be prescribed only by licensed and experienced health care providers after a thorough history and physical examination have been performed. The side‐effects of the medications must be explained, and the patient should be advised to seek medical attention if a side‐effect occurs and to discontinue the drug if the side‐effect is deemed serious. Patients must be informed that some of the side‐effects may persist even when finasteride has been discontinued [48]. These drugs have the potential to markedly help with hair regrowth. However, they must be prescribed or recommended responsibly, and their usage monitored.

## 5 Conclusion

In this network meta‐analysis, dutasteride at a daily dose of 0.5 mg emerged as the most effective overall treatment for androgenetic alopecia (AGA). Among FDA‐approved therapies, topical minoxidil 5% was identified as the most effective topical agent, while oral finasteride 1 mg/day was the most effective oral option. Notably, mesotherapy with dutasteride was significantly less effective than oral administration of dutasteride 0.5 mg/day.

Oral dutasteride 0.5 mg/day demonstrated superior efficacy compared to both oral finasteride 1 mg/day and oral minoxidil 5 mg/day. Various formulations of minoxidil—including oral (5 mg/day), topical (5% twice daily and 2% twice daily), and sublingual (5 mg once daily)—showed comparable efficacy in the treatment of AGA. This similarity may be due, in part, to limited sample sizes in the underlying studies, which could preclude detection of subtle differences in treatment response.

Based on available data, oral finasteride 1 mg/day and topical finasteride 0.25% appear to offer similar efficacy. However, data on the efficacy of topical finasteride, particularly in North America, remain limited, and it is not currently FDA‐approved to treat AGA.

## Author Contributions

Conception of the manuscript was done by A.K.G. Data analysis was performed by M.A.B. The manuscript was drafted by A.K.G., M.T., G.W., and M.A.B. substantively edited and revised by A.K.G., M.T., G.W., and M.A.B.

## Ethics Statement

The authors have nothing to report.

## Conflicts of Interest

The authors declare no conflicts of interest.

## Supporting information


**FIGURE S1.** League table for 24‐week change in total hair density (17 interventions).

## Data Availability

Data can be made available upon reasonable request.
