# Supplementary material for: Comparative Efficacy of Minoxidil and 5‐Alpha Reductase Inhibitors Monotherapy for Male Pattern Hair Loss: Network Meta‐Analysis Study of Current Empirical Evidence
Source: J Cosmet Dermatol. 2025 Jun 30;24(7):e70320. doi: 10.1111/jocd.70320 (PMC12207719; doi:10.1111/jocd.70320)
Supplement: Supplementary file 1 — FIGURE S1. League table for 24‐week change in total hair density (17 interventions). [file JOCD-24-e70320-s001.pdf]

- *p* <0.05 (highlighted cells, shown in bold)
- U.S. FDA Approved
- Approved in Italy, Germany, Portugal, Switzerland, Spain, South Korea, Saudi Arabia
- Approved in Japan, Taiwan, Korea
- Off-label treatment options

**DUTA A (Oral)**																	
2.4 (-3.5, 8.4)	**FIN A (Oral)**																
4.8 (-2.3, 12)	2.4 (-6.4, 11.3)	**MX A (Top)**															
5.2 (-1.8, 12.2)	2.8 (-6, 11.5)	0.4 (-2.3, 3)	**FIN A (Top)**														
**6.2 (3.8, 8.6)**	3.8 (-2.2, 9.8)	1.4 (-5.8, 8.5)	1 (-6.1, 8)	**DUTA B (Oral)**													
**7 (5, 9.2)**	4.7 (-1.1, 10.4)	2.2 (-4.7, 9.2)	1.9 (-5, 8.7)	0.8 (-1.4, 3.2)	**FIN B (Oral)**												
**7.5 (0.9, 14)**	5.1 (-3.3, 13.5)	2.7 (-6.7, 11.9)	2.3 (-6.9, 11.4)	1.3 (-5.3, 7.9)	0.4 (-5.9, 6.7)	**FIN C (Oral)**											
**7.8 (4.4, 11.4)**	5.4 (-1, 11.9)	3 (-4.5, 10.4)	2.7 (-4.8, 10)	1.6 (-2, 5.3)	0.8 (-2, 3.6)	0.4 (-6.6, 7.2)	**FIN B (Top)**										
**8.5 (4.8, 12.3)**	6.2 (-0.1, 12.5)	3.7 (-3.7, 11)	3.4 (-3.9, 10.5)	2.4 (-1.4, 6.1)	1.5 (-2, 5)	1.1 (-5.9, 8.1)	0.7 (-3.8, 5.2)	**MX B (Top)**									
**9.2 (5.9, 12.6)**	**6.8 (0.7, 12.9)**	4.4 (-3, 1..8)	4 (-3.3, 11.3)	3 (-0.4, 6.5)	2.2 (-1.1, 5.3)	1.7 (-5.1, 8.5)	1.4 (-2.9, 5.6)	0.6 (-3.3, 4.7)	**DUTA (Meso)**								
10.9 (-17.8, 39.7)	8.5 (-20.7, 37.7)	6.2 (-23.3, 35.5)	5.7 (-23.7, 35.2)	4.8 (-24.1, 33.5)	3.9 (-24.9, 32.5)	3.5 (-25.8, 33)	3.1 (-25.8, 31.9)	2.4 (-26.3, 30.9)	1.8 (-27.2, 30.5)	**MX (Subl)**							
**11 (3.4, 18.6)**	8.6 (-0.7, 17.8)	**6.1 (1.7, 10.7)**	**5.8 (0.7, 10.9)**	4.8 (-2.8, 12.5)	3.9 (-3.5, 11.4)	3.5 (-6.1, 13.2)	3.2 (-4.7, 11.2)	2.4 (-5.3, 10.2)	1.8 (-6, 9.6)	0 (-29.3, 29.4)	**MX C (Top)**						
**13.9 (3.4, 24.4)**	11.6 (-0.2, 23.2)	9.1 (-2.9, 21)	8.8 (-3.3, 20.6)	7.8 (-2.8, 18.2)	6.9 (-17.2, 3.6)	6.5 (-5.6, 18.4)	6.1 (-4.7, 16.8)	5.4 (-4.6, 15.2)	4.8 (-5.9, 15.3)	3 (-23.7, 29.9)	3 (-9.3, 15.1)	**MX A (Oral)**					
**14.4 (7.5, 21.4)**	**12 (3.4, 20.7)**	**9.6 (0.3, 18.9)**	**9.3 (0, 18.4)**	**8.3 (1.3, 15.3)**	**7.4 (0.5, 14.2)**	7 (-2.2, 16.1)	6.6 (-0.8, 14)	5.9 (-0.1, 11.8)	5.2 (-1.9, 12.3)	3.5 (-24.4, 31.6)	3.5 (-6.1, 13)	0.5 (-7.7, 8.7)	**MX D (Top)**				
**14.3 (12, 16.8)**	**12 (6, 17.9)**	**9.6 (2.4, 16.7)**	**9.2 (2.1, 16.2)**	**8.2 (5.7, 10.6)**	**7.3 (5, 9.5)**	**6.9 (0.3, 13.5)**	**6.6 (2.9, 10.2)**	**5.8 (2.1, 9.6)**	**5.2 (1.7, 8.6)**	3.4 (-25.3, 32.3)	3.4 (-4.3, 11)	0.4 (-10, 11.1)	-0.1 (-7.1, 6.9)	**DUTA C (Oral)**			
**18.8 (16.7, 21)**	**16.4 (10.9, 21.9)**	**14 (7.1, 20.9)**	**13.7 (6.8, 20.4)**	**12.6 (10.4, 14.9)**	**11.8 (9.9, 13.5)**	**11.3 (5, 17.7)**	**11 (7.6, 14.3)**	**10.3 (7.2, 13.3)**	**9.6 (7, 12.2)**	7.9 (-20.8, 36.6)	**7.9 (0.4, 15.2)**	4.8 (-5.3, 15.2)	4.4 (-2.2, 11)	**4.4 (2.1, 6.7)**	**Control**		
**23.1 (8.7, 37.4)**	**20.8 (5.3, 36)**	**18.4 (2.1, 34)**	**18 (1.9, 33.6)**	**16.9 (2.5, 31.2)**	**16.1 (1.7, 30.4)**	**15.7 (0, 31.2)**	**15.3 (0.6, 29.8)**	14.6 (-0.1, 29.2)	13.9 (-0.7, 28.4)	12.1 (-19.6, 44.1)	12.2 (-4.2, 28.1)	9.2 (-8.5, 26.7)	8.7 (-7.2, 24.4)	8.8 (-5.7, 23)	4.3 (-10.1, 18.5)	**MX B (Oral)**	
**21.8 (11.9, 32)**	**19.5 (8.2, 30.8)**	**17 (6.2, 28.1)**	**16.7 (5.8, 27.8)**	**15.7 (5.7, 25.9)**	**14.8 (4.9, 25)**	**14.4 (2.8, 26.3)**	**14.1 (3.8, 24.5)**	**13.3 (3.4, 23.5)**	**12.7 (2.6, 23)**	10.9 (-19.1, 41.4)	**10.9 (0.1, 21.8)**	8 (-5.9, 22)	7.5 (-4.1, 19.1)	7.5 (-2.5, 17.7)	3.1 (-6.7, 13.1)	-1.2 (-18.5, 16.3)	**MX E (Top)**

Supplementary Figure 1: League table for 24-week change in total hair density (17 interventions). This league table presents, for the largest network, pairwise relative effects for selected interventions. Herein, relative effects have been quantified in terms of the mean difference (MD) and its corresponding 95% credible interval (CI) is presented in parentheses. Control corresponds to placebo/vehicle and the abbreviations for the selected active comparators are as follows:

DUTA A (Oral): Dutasteride (oral) 0.5 mg/ day, DUTA B (Oral): Dutasteride (oral) 0.1 mg/ day, DUTA C (Oral): Dutasteride (oral) 0.02 mg/day. DUTA (Meso): Dutasteride (mesotherapy) 0.05%, FIN A (Oral): Finasteride (oral) 5 mg/day, FIN B (Oral): Finasteride (oral) 1 mg/day, FIN C (Oral): Finasteride (oral) 0.2 mg/day, FIN A (Top): Finasteride (topical) 0.25% once or twice daily , FIN B (Top): Finasteride (topical) 1% twice daily, MX A (Oral): Minoxidil (oral) 5 mg/day, MX B (Oral): Minoxidil (oral) 0.25 mg/day, MX A (Top): Minoxidil (topical) 5% twice daily, MX B (Top): Minoxidil (topical) 2% twice daily, MX C (Top): Minoxidil (topical) 1% twice daily, MX D (Top): Minoxidil (topical) 3% twice daily, MX E (Top): Minoxidil (topical) 0.1% twice daily, MX (Subl): Minoxidil (sublingual) 5 mg/day
